# Quantitative Assessment of the Influence of Cytochrome P450 1A2 Gene Polymorphism and Colorectal Cancer Risk

**DOI:** 10.1371/journal.pone.0071481

**Published:** 2013-08-12

**Authors:** Yuan Zhao, Zi-Xian Chen, Abudouaini Rewuti, Yu-Shui Ma, Xiao-Feng Wang, Qing Xia, Da Fu, Yu-Song Han

**Affiliations:** 1 Department of Gastroenterology, Zhongshan Hospital, Fudan University, Shanghai, People’s Republic of China; 2 Department of Orthopedics Surgery, Zhongshan Hospital, Fudan University, Shanghai, People’s Republic of China; 3 Department of General Surgery, Zhongshan Hospital, Fudan University, Shanghai, People’s Republic of China; The University of Texas M. D. Anderson Cancer Center, United States of America

## Abstract

Cytochrome P450 1A2 (*CYP1A2*) encodes a member of the cytochrome P450 superfamily of enzymes, which play a central role in activating and detoxifying many carcinogens and endogenous compounds thought to be involved in the development of colorectal cancer (CRC). The CYP1A2*C (rs2069514) and CYP1A2*F (rs762551) polymorphism are two of the most commonly studied polymorphisms of the gene for their association with risk of CRC, but the results are conflicting. To derive a more precise estimation of the relationship between *CYP1A2* and genetic risk of CRC, we performed a comprehensive meta-analysis which included 7088 cases and 7568 controls from 12 published case-control studies. In a combined analysis, the summary per-allele odds ratio for CRC was 0.91 (95% CI: 0.83–1.00, P = 0.04), and 0.91 (95% CI: 0.68–1.22, P = 0.53), for *CYP1A2* *F and *C allele, respectively. In the subgroup analysis by ethnicity, significant associations were found in Asians for CYP1A2*F and CYP1A2*C, while no significant associations were detected among Caucasian populations. Similar results were also observed using dominant genetic model. Potential sources of heterogeneity were explored by subgroup analysis and meta-regression. No significant heterogeneity was detected in most of comparisons. This meta-analysis suggests that the *CYP1A2* *F and *C polymorphism is a protective factor against CRC among Asians.

## Introduction

Given its high prevalence and poor prognosis, colorectal cancer (CRC) is very much a public health issue in industrialized Western countries. In the European population it comprised 13.4% of all newly diagnosed carcinomas in 2008 [1]. Most CRCs develop through multiple mutations in the normal colonic mucosa and evolve through the adenoma-carcinoma sequence [2], [3]. Development of sporadic colorectal adenomas and carcinomas has been associated with several lifestyle factors, including cigarette smoking [4], [5] and dietary items such as red meat [6], [7].

Cigarette smoke is a major source of a wide variety of carcinogens, including nitrosamines, polycyclic hydrocarbons (PAHs), aromatic amines (AAs) and heterocyclic aromatic amines (HCAs) [8], [9]. Carcinogens in cigarettes may reach the colorectal mucosa through the circulatory system [10]. Long-term, heavy cigarette smokers have a 2- to 3-fold elevated risk of colorectal adenoma and the vast majority of studies in the past several years show an association between cigarette use and CRC [4]. Carcinogens that form during the cooking or processing of meats have been postulated as potential culprits for the association between red meats and CRC risk. These include: HCAs, PAHs and N-nitroso compounds (NOCs) [11]. High cooking temperature or prolonged duration of cooking favors the formation of HCA [12]. A few epidemiological studies have considered estimated levels of HCAs from diets high in well-done red meat and overall support a role for HCAs in CRC [13], [14]. Exposure to NOCs can occur from exogenous sources, such as cured meats with nitrites, or from endogenous formation due to nitrosating agents that react with amines derived from red meat [15]. However, the relative contribution of each of these carcinogens to CRC is still uncertain.

The influence of exposures on CRC development may be affected by variation in biotransformation of carcinogens. The cytochrome P450 (CYP)-dependent monooxygenase (Phase I enzyme) represents the first line of defense against toxic chemicals. CYP1A2 is the major enzyme involved in the metabolism of HCAs and AAs [16] and phenotype studies have detected large inter-individual variation of *CYP1A2* expression in the liver [17]. In addition, variation in CYP1A2 activity in humans may be due to various environmental exposures, including cigarette smoke [18], genetic differences [19] or gene-gene interaction [20]. Two polymorphisms of the *CYP1A2* gene, CYP1A2*1C (3858G→A) and CYP1A2*1F (164A→C), have been examined to associate with reduced enzyme activity [18], [21]. Associations between the two polymorphisms and CRC have been independently replicated by many studies [22]–[33]; however, a proportion of them have produced contrary results. These disparate findings may be due partly to insufficient power, ethnic diversity and publication biases. We therefore performed a meta-analysis of the published studies to clarify this inconsistency and to establish a comprehensive picture of the relationship between *CYP1A2* and CRC.

## Materials and Methods

### Literature Search Strategy and Inclusion Criteria

Papers published before the end of Dec. 2012 were identified through a search of Pubmed, ISI Web of Science and Embase using the following terms “colorectal” or “colo*,” “cancer” or “tumor” or “carcinoma,” and “*CYP1A2*” or “cytochrome P450 1A2”, without restriction on language. All references cited in these studies and previously published review articles were reviewed to identify additional eligible studies. Only those studies assessing the association between the CRC and the *CYP1A2* gene polymorphisms were included. The inclusion criteria were (1) original papers containing independent data, (2) identification of CRC was confirmed pathologically or histologically, (3) sufficient data to calculate the odds ratio (OR) or P-value and (4) case–control or cohort studies. The major reasons for exclusion of studies were (1) overlapping data and (2) case-only studies and review articles.

### Data Extraction

For each study, the following information was extracted independently by two investigators: first author’s surname, publication date, gender, ethnicity, genotyping method, cigarette smoking status, age, sex, confirmation of diagnosis, Hardy–Weinberg equilibrium (HWE) status, and genotype frequency in cases and controls. The results were compared and disagreements were discussed and resolved with consensus. Where essential information was not presented in articles, every effort was made to contact the authors.

### Statistical Methods

The strength of the association between *CYP1A2* polymorphisms and CRC risk was evaluated by the odds ratios (ORs) with 95% confidence intervals (CIs). The per-allele odds ratio (OR) of the risk allele was compared between cases and controls. Then we examined the association between risk genotype of the polymorphism and CRC susceptibility using dominant model. HWE in the control group was assessed using Fisher’s exact test.

Cochran’s X^2^ based Q-statistic [34] test and I^2^-test [35] was performed to assess possible heterogeneity in the combined studies. If heterogeneity existed, the random effects model (the DerSimonian and Laird method) [36], which yields wider confidence intervals, was adopted to calculate the overall OR value. Otherwise, the fixed effects model (the Mantel–Haenszel method) was used [37]. In addition, sources of heterogeneity were investigated by stratified meta-analyses based on ethnicity (Caucasian and Asian population), source of controls (population and hospital based), sample size (No. cases ≥500 or <500). The significance of the overall OR was determined by the Z-test. Funnel plots and Egger’s linear regression test were used to assess evidence for potential publication bias [38]. In order to assess the stability of the result, sensitivity analyses were performed, each study in turn was removed from the total, and the remaining were reanalyzed. The analyses were carried out by using the Stata software version 10.0 (Stata Corporation, College Station, TX). The type I error rate was set at 0.05. All P-values were two-tailed.

## Results

### Characteristics of Studies

The combined search yielded 85 references. Seventy-three articles were excluded because they clearly did not meet the criteria or overlapping references (Figure S1). Finally, a total of 12 studies with 7088 cases and 7568 controls examined the association between the *CYP1A2* polymorphism and CRC were included in the current meta-analysis [22]–[33]. Among them, 11 studies were identified for the *CYP1A2* *F polymorphism, including a total of 6370 cases and 6837 controls, and for the *CYP1A2* *C polymorphism 5 studies were identified covering a total of 1283 cases and 1205 controls. The genotype distributions in the controls for all studies were consistent with HWE. Characteristics of studies included in the current meta-analysis are presented in Table 1.

**Table 1:** Characteristics of the studies included in the meta-analysis.

Reference	Year	Ethnicity (Ethnic group)	Case	Polymorphism	No. of case/control	Mean age ofcase/control	Sex in case/control (male%)	Source ofcontrol	Genotyping method
Wang [22]	2012	American (Caucasian)	Colonoscopy confirmed	CYP1A2*F	570/357	60.0/59.3	52.3/46.3	Population	TaqMan
Sainz [23]	2011	German (Caucasian)	ICD-10: C18–C20	CYP1A2*F	1764/1786	NA/NA	NA/NA	Population	KASPar
Cleary [24]	2010	Canadian (Caucasian)	ICD-9 classification 153.0–153.9, 154.0–154.1	CYP1A2*F	1165/1290	NA/NA	41.0/56.0	Population	TaqMan
Wang [25]	2010	American (Caucasian)	Histologically confirmed	CYP1A2*F	496/607	66.0/67.0	61.5/58.1	Population	TaqMan
Yeh [26]	2009	Chinese (Asian)	Histologically confirmed	CYP1A2*C	718/631	60.3/60.7	56.4/55.6	Population	PCR–RFLP
Saebø [27]	2008	Norwegian (Caucasian)	CRC patients	CYP1A2*F	198/222	67.7/54.8	53.0/41.0	Population	PCR–RFLP
Küry [28]	2007	French (Caucasian)	CRC patients	CYP1A2*F	1013/1118	67.0/62.0	62.0/54.0	Population	TaqMan
Yoshida [29]	2007	Japanese (Asian)	CRC patients	CYP1A2*F, CYP1A2*C	64/111	67.3/67.3	54.5/60.3	Hospital	PCR–RFLP
Bae [30]	2006	Korean (Asian)	Colonoscopy confirmed	CYP1A2*F, CYP1A2*C	111/93	62.5/49.2	54.1/59.1	Hospital	PCR–RFLP
Chen [31]	2005	Chinese (Asian)	CRC patients	CYP1A2*F	138/340	58.8/58.5	49.3/47.2	Population	PCR–RFLP
Landi [32]	2005	Spanish (Caucasian)	CRC patients	CYP1A2*F, CYP1A2*C	361/321	NA/NA	NA/NA	Hospital	APEX
Sachse [33]	2002	British (Caucasian)	ICD-9 classification 153.0–153.9, 154.0–154.1	CYP1A2*F, CYP1A2*C	490/592	67.7/68.6	61.0/54.0	Population	PCR–RFLP

ICD: International Classification of Diseases; NA: Not Available.

### CYP1A2*F and CRC Risk

**Figure 1:**
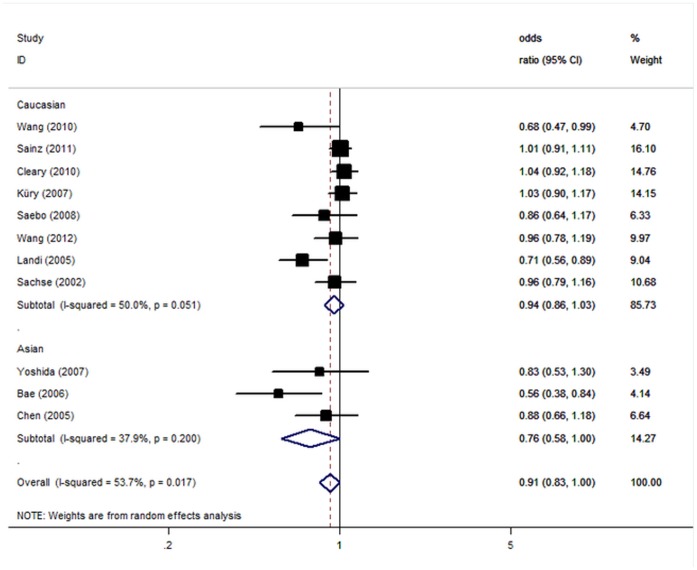
Forest plot from the meta-analysis of CYP1A2*F polymorphism and CRC risk.

Significant heterogeneity was present among the 11 studies of the CYP1A2*F polymorphism (P = 0.01). Ethnicity (P = 0.02) and sample size (P = 0.01) explained a large part of the heterogeneity, whereas source of controls (P = 0.21), mean age of cases (P = 0.51) and controls (P = 0.14), and sex distribution of cases (P = 0.53) and controls (P = 0.99) explained little heterogeneity. Using random effect model, the per-allele overall OR of the A variant for CRC was 0.91 (95% CI: 0.83–1.00, P = 0.04; figure 1) with corresponding results under dominant genetic model of 0.97 (95% CI: 0.89–1.07, P = 0.68).

In the stratified analysis by ethnicity, significant associations were detected among Asians in all genetic models (allele contrast: OR = 0.76, 95% CI: 0.58–1.00; dominant model: OR = 0.89, 95% CI: 0.77–0.99). However, we failed to detect any association to CRC risk for Caucasians in all genetic models. By considering control source subgroups, the OR was 0.99 (95% CI: 0.93–1.05, P = 0.71) in population-based controls compared to 0.69 (95% CI: 0.58–0.83, P<10^−4^) in hospital controls. Subsidiary analyses of sample size yielded a per-allele OR for small studies of 0.80 (95% CI: 0.70–0.91, P = 0.001), while no significant results were found for large studies (Table 2).

**Table 2:** Main results of overall and subgroups in the meta-analysis.

Polymorphism	Sub-group analysis	Allele contrast				Dominant model			
		OR (95% CI)	P(Z)	P(Q)	I^2^	OR (95% CI)	P(Z)	P(Q)	I^2^
CYP1A2*F (rs762551)	Total	0.91 (0.83–1.00)	0.04	0.02	53.7%	0.97 (0.89–1.07)	0.68	0.58	50.9%
	Ethnicity								
	Caucasian	0.94 (0.86–1.03)	0.20	0.05	50.0%	0.98 (0.84–1.11)	0.58	0.41	47.3%
	Asian	0.76 (0.58–1.00)	0.04	0.20	37.9%	0.89 (0.77–0.99)	0.04	0.54	35.0%
	Control source								
	Population	0.99 (0.93–1.05)	0.71	0.48	26.8%	1.00 (0.88–1.15)	0.97	0.94	0.0%
	Hospital	0.69 (0.58–0.83)	<10^−4^	0.44	38.4%	0.86 (0.67–0.98)	0.01	0.13	17.0%
	Sample size								
	<500	0.80 (0.70–0.91)	0.001	0.20	11.6%	0.74 (0.57–0.97)	0.03	0.42	0.0%
	≥500	1.02 (0.95–1.08)	0.63	0.93	0.0%	1.01 (0.88–1.17)	0.58	0.71	0.0%
CYP1A2*C (rs2069514)	Total	0.91 (0.68–1.22)	0.53	0.14	42.2%	0.84 (0.59–1.21)	0.35	0.13	46.1%
	Ethnicity								
	Caucasian	2.84 (0.24–34.23)	0.41	0.03	80.2%	2.85 (0.20–40.79)	0.44	0.02	66.3%
	Asian	0.84 (0.72–0.97)	0.02	0.74	0.0%	0.78 (0.65–0.94)	0.01	0.86	0.0%
	Control source								
	Population	2.53 (0.19–33.30)	0.48	0.01	45.5%	2.59 (0.17–38.81)	0.49	0.009	73.8%
	Hospital	0.89 (0.66–1.22)	0.48	0.75	0.0%	0.76 (0.53–1.11)	0.16	0.83	0.0%

P(Z): Z test used to determine the significance of the overall OR; P(Z)<0.05 was considered statistically significant.

P(Q): Cochran’s chi-square Q statistic test used to assess the heterogeneity between-studies; P(Q)<0.05 was considered statistically significant.

A funnel plot of these 11 studies suggested a possibility of the preferential publication of positive findings in smaller studies (Egger test, P = 0.03, Figure S2). Analysis restricted to the 4 studies with at least 500 cases (total, 4512 cases and 4551 controls), which should be less prone to selective publication than smaller studies, yielded an OR of 1.02 (95% CI: 0.95–1.08, P = 0.63). No heterogeneity was present among the 4 studies of the CYP1A2*F polymorphism (P = 0.93). Sensitivity analysis indicated that no single study influenced the pooled OR qualitatively, suggesting that the results of this meta-analysis are stable.

### CYP1A2*C and CRC Risk

**Figure 2:**
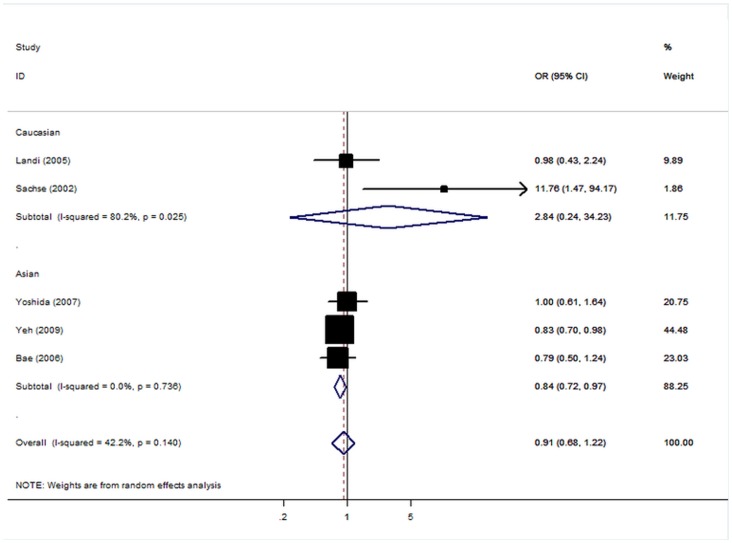
Forest plot from the meta-analysis of CYP1A2*C polymorphism and CRC risk.

In the overall analysis, the risk G allele CYP1A2*C was not significantly associated with elevated risk of CRC (Figure 2). When studies were stratified for ethnicity, significant risks were found among Asians in all genetic model (G allele: OR = 0.84, 95% CI: 0.72–0.97, P = 0.02; dominant model: OR = 0.78, 95% CI: 0.65–0.94, P = 0.01). However, no significant association was found for Caucasian populations in all genetic models. Further stratified according to source of controls, no significant results were found in all genetic models (Table 2).

The shape of the funnel plot did not indicate any evidence of obvious asymmetry (Figure S3), thus suggesting no publication bias among the studies included. Egger’s test was used to provide further statistical evidence; similarly, the results showed no significant publication bias in this meta-analysis (Egger test, P = 0.14).

## Discussion

This is the first comprehensive meta-analysis examined the polymorphisms of *CYP1A2* and genetic susceptibility to CRC. In total, the meta-analysis involved 12 studies for CRC including 7088 cases and 7568 controls. Our results demonstrated CYP1A2*F polymorphism is a protective factor against CRC. Besides, significant association was also detected for the CYP1A2*C polymorphism among Asians. As the sample size was considerably smaller for Asian studies, so the results must be interpreted with caution. Such result could be due to limited number of studies that had insufficient statistical power to detect a slight effect or may have generated a fluctuated risk estimate. Therefore, larger studies of different ethnic populations, especially among Asians, are needed to confirm our findings.

In meta-analysis, heterogeneity evaluation was always conducted in statistical analysis. Thus, several subgroup meta-analyses were performed according to ethnicity, sample size, and control source. After stratified by ethnicity, significant association was observed among Asians, but not among Caucasians, a possible reflection of differences in genetic background and gene–environment interactions in the etiology. In fact, the distribution of the less common *F allele varies extensively between different races, with a prevalence of ∼35% among Asians, and ∼27% among Caucasians, suggesting a possible ethnic difference. On the other hand, it is possible that variation at this locus has modest effects on CRC, but environmental factors may predominate in the progress of CRC, and mask the effects of this variation. Specific environmental factors like lifestyle and cigarette smoking have already been well studied in recent decades [28]. The unconsidered factors mixed together may cover the role of *CYP1A2* polymorphism. Thus, even if the variation has a causal effect on colorectal cancer, it may take a long time to be observed. Finally, different populations usually have different linkage disequilibrium patterns. A polymorphism may be in close linkage with another nearby causal variant in one ethnic population but not in another. In the stratified analysis by control source, we found significant associations between CYP1A2*F carriers and CRC risk for detected in hospital-based studies but not in population-based studies. This reason may be that the hospital-based studies have some biases because such controls may just represent a sample of ill-defined reference population, and may not be representative of the general population very well, particularly when the genotypes under investigation were associated with the disease conditions that the hospital-based controls may have. Therefore, using a proper and representative population-based control subjects is very important to reduce biases in such genetic association studies.

CYP1A2 is an inducible phase I metabolizing enzyme which plays a key role in the metabolism of HCAs [39]. The CYP1A2*F (164A→C) polymorphism is common among Caucasians [21] and it may explain the reported variation in *CYP1A2* inducibility [19]. The A allele is associated with higher enzymatic activity compared with the protein coded by the C allele [19]. Therefore, an effect modification of this SNP on the effect of HCAs on CRC risk is plausible. However, in which way the C allele affects inducibility and enzyme activity is not clear. Studies of the CYP1A2*F polymorphism and protein activity in humans have reported conflicting evidence. Both the A/A and any C allele had either no effect [40]–[42], or increased, or decreased activity [19], [21], [30], [43]. Various markers have been used to assess protein activity (urinary caffeine metabolites, plasma metabolic ratio, urinary PhIP metabolites, clozapine serum concentrations) which makes it difficult to compare results from different studies. A Korean study used the urinary caffeine challenge test to analyze the genotype phenotype association and found that the CYP1A2 activity in healthy smokers with the C allele was significantly higher than that in individuals with the A/A genotype [30]. The genotype frequencies of the CYP1A2*F polymorphism in the Korean study [30] were comparable to the result in this study and other Caucasian studies [19], [33]. To clarify the effect of CYP1A2*F polymorphism on activity, identical methods for measuring activity should be used in additional studies to enhance our understanding of the genotype–phenotype associations. The CYP1A2*F polymorphism is located in intron 1 and variation in activity may be due to both environmental exposures and gene–gene interactions [20]. Unfortunately, very few of included studies explore the interaction between *CYP1A2* genotype and environmental risk factor exposure such as smoking habits. This was probably due to the low statistical power of the individual studies to detect interactions. For, CYP1A2*C the functional significance of the CYP1A2*C allele remains unclear. Some studies found decreased enzyme activity or inducibility associated with the A allele [18], [44]. Other studies found no difference in the enzyme activities or inducibility between the G and A alleles [21], [45]. One study reported the A allele was related to increased CYP1A2 activity [46]. Recently, Wang et al. [47] reported a meta-analysis and found no association between CYP1A2*F and genetic susceptibility to cancer among Asians. However, the Asian population reports in the study include a mixture of various types of cancer. As cancer is a complex and heterogeneous disease, different types of cancer may have different biologic mechanisms that underpin tumor heterogeneity. Thus, the effect of single genetic factor on the risk of cancer may be more pronounced in the presence of other common genetic or environmental risk factors such as smoking, hepatitis virus infection. In addition, such result could be due to limited number of included studies that had insufficient statistical power to detect a slight effect. In the present study, we focused on *CYP1A2* and genetic susceptibility to CRC which significantly decreased tumor heterogeneity. Furthermore, we explored potential sources of heterogeneity across studies. Besides, our results suggest an overestimation of the true genetic association by small studies, consistent with the phenomenon known as ‘winner’s curse’ [48], [49].

Several limitations of this meta-analysis should be addressed. Firstly, our results were based on unadjusted estimates, while a more precise analysis should be conducted if all individual raw data were available, which would allow for the adjustment by other co-variants including age, drinking status, cigarette consumption and other lifestyle. Secondly, the sample size was still relatively small for the stratified analysis. Thirdly, most of the included studies have conducted on Caucasians and a few on Asians, so that the results must be interpreted with caution. Further studies concerning populations in other areas are required to diminish the ethnic variation produced biases.

In conclusion, this meta-analysis suggested that the CYP1A2*C and CYP1A2*F polymorphism was associated with decreased CRC risk for Asian populations. It is also known that the pathogenesis of CRC is complex and polygenetic in the vast majority of patients, with several genes, each with a small to moderate effect, acting individually, together or in association with important environmental determinants. Larger studies of different ethnic populations, especially with detailed individual information, are needed to confirm our findings.

## Supporting Information

Figure S1 **Flow chart of literature search for studies examining** ***CYP1A2*** **gene polymorphism and risk of CRC.**
(TIF)

Figure S2 **Funnel plot of studies of the** ***CYP1A2*** *F polymorphism and CRC showing a possible excess of smaller studies with strikingly positive findings beyond the 95% CI.
(TIF)

Figure S3 **Funnel plot for the association between and** ***CYP1A2*** *C and CRC risk; Egger’s test was also performed to investigate the symmetry of the funnel plot ( ***P*** **= 0.14).**
(TIF)

Checklist S1 (DOC)

## References

[1] Ferlay J, Shin HR, Bray F, Forman D, Mathers C, et al (2010) Estimates of worldwide burden of cancer in 2008: GLOBOCAN 2008. Int J Cancer 127: 2893–2917. 21351269 10.1002/ijc.25516

[2] Vogelstein B, Fearon ER, Hamilton SR, Kern SE, Preisinger AC, et al (1988) Genetic alterations during colorectal-tumor development. N Engl J Med 319: 525–532. 2841597 10.1056/NEJM198809013190901

[3] Bond JH (2000) Clinical evidence for the adenoma carcinoma sequence and the management of patients with colorectal adenomas. Semin Gastrointest Dis 11: 176–184. 11057945

[4] Giovannucci E (2001) An updated review of the epidemiological evidence that cigarette smoking increases risk of colorectal cancer. Cancer Epidemiol Biomarkers Prev 10: 725–731. 11440957

[5] Almendingen K, Hofstad B, Trygg K, Hoff G, Hussain A, et al (2000) Smoking and colorectal adenomas: a case-control study. Eur J Cancer Prev 9: 193–203. 10954259

[6] Heavey PM, McKenna D, Rowland IR (2004) Colorectal cancer and the relationship between genes and the environment. Nutr Cancer 48: 124–141. 15231447 10.1207/s15327914nc4802_2

[7] Potter JD (1999) Colorectal cancer: molecules and populations. J Natl Cancer Inst 91: 916–932. 10359544 10.1093/jnci/91.11.916

[8] Manabe S, Tohyama K, Wada O, Aramaki T (1991) Detection of a carcinogen 2-amino-1-methyl-6-phenylimidazo[45-b]pyridine (PhIP) in cigarette smoke condensate. Carcinogenesis 12: 1945–1947. 1934275 10.1093/carcin/12.10.1945

[9] Wogan GN, Hecht SS, Felton JS (2004) Environmental and chemical carcinogenesis. Semin Cancer Biol 14: 473–486. 15489140 10.1016/j.semcancer.2004.06.010

[10] Kune GA, Kune S, Vitetta L, Watson LF (1992) Smoking and colorectal cancer risk: data from the Melbourne Colorectal Cancer Study and brief review of literature. Int J Cancer 50: 369–372. 1735604 10.1002/ijc.2910500307

[11] Cross AJ, Sinha R (2004) Meat-related mutagens/carcinogens in the etiology of colorectal cancer. Environ Mol Mutagen 44: 44–55. 15199546 10.1002/em.20030

[12] Sugimura T (1985) Carcinogenicity of mutagenic heterocyclic amines formed during the cooking process. Mutat Res 150: 33–41. 3889618 10.1016/0027-5107(85)90098-3

[13] Butler LM, Sinha R, Millikan RC, Martin CF, Newman B, et al (2003) Heterocyclic amines, meat intake, and association with colon cancer in a population-based study. Am J Epidemiol 157: 434–445. 12615608 10.1093/aje/kwf221

[14] Cross AJ, Ferrucci LM, Risch A, Graubard BI, Ward MH, et al (2010) A large prospective study of meat consumption and colorectal cancer risk: an investigation of potential mechanisms underlying this association. Cancer Res 70: 2406–2414. 20215514 10.1158/0008-5472.CAN-09-3929 PMC2840051

[15] Hughes R, Cross AJ, Pollock JR, Bingham S (2001) Dose-dependent effect of dietary meat on endogenous colonic N-nitrosation. Carcinogenesis 22: 199–202. 11159760 10.1093/carcin/22.1.199

[16] Eaton DL, Gallagher EP, Bammler TK, Kunze KL (1995) Role of cytochrome P4501A2 in chemical carcinogenesis: implications for human variability in expression and enzyme activity. Pharmacogenetics 5: 259–274. 8563766 10.1097/00008571-199510000-00001

[17] Aitchison KJ, Gonzalez FJ, Quattrochi LC, Sapone A, Zhao JH, et al (2000) Identification of novel polymorphisms in the 5′ flanking region of CYP1A2 characterization of interethnic variability and investigation of their functional significance. Pharmacogenetics 10: 695–704. 11186132 10.1097/00008571-200011000-00004

[18] Nakajima M, Yokoi T, Mizutani M, Kinoshita M, Funayama M, et al (1999) Genetic polymorphism in the 5′-flanking region of human CYP1A2 gene: effect on the CYP1A2 inducibility in humans. J Biochem 125: 803–808. 10101295 10.1093/oxfordjournals.jbchem.a022352

[19] Sachse C, Brockmoller J, Bauer S, Roots I (1999) Functional significance of a C–>A polymorphism in intron 1 of the cytochrome P450 CYP1A2 gene tested with caffeine. Br J Clin Pharmacol 47: 445–449. 10233211 10.1046/j.1365-2125.1999.00898.x PMC2014233

[20] MacLeod S, Sinha R, Kadlubar FF, Lang NP (1997) Polymorphisms of CYP1A1 and GSTM1 influence the in vivo function of CYP1A2. Mutat Res 376: 135–142. 9202749 10.1016/s0027-5107(97)00036-5

[21] Sachse C, Bhambra U, Smith G, Lightfoot TJ, Barrett JH, et al (2003) Polymorphisms in the cytochrome P450 CYP1A2 gene (CYP1A2) in colorectal cancer patients and controls: allele frequencies, linkage disequilibrium and influence on caffeine metabolism. Br J Clin Pharmacol 55: 68–76. 12534642 10.1046/j.1365-2125.2003.01733.x PMC1884179

[22] Wang J, Joshi AD, Corral R, Siegmund KD, Marchand LL, et al (2012) Carcinogen metabolism genes, red meat and poultry intake, and colorectal cancer risk. Int J Cancer 130: 1898–1907. 21618522 10.1002/ijc.26199 PMC3883510

[23] Sainz J, Rudolph A, Hein R, Hoffmeister M, Buch S, et al (2011) Association of genetic polymorphisms in ESR2, HSD17B1, ABCB1, and SHBG genes with colorectal cancer risk. Endocr Relat Cancer 18: 265–276. 21317201 10.1530/ERC-10-0264

[24] Cleary SP, Cotterchio M, Shi E, Gallinger S, Harper P (2010) Cigarette Smoking, Genetic Variants in Carcinogen-metabolizing Enzymes, and Colorectal Cancer Risk. Am J Epidemiol 172: 1000–1014. 20937634 10.1093/aje/kwq245 PMC2984254

[25] Wang H, Yamamoto JF, Caberto C, Saltzman B, Decker R, et al (2010) Genetic variation in the bioactivation pathway for polycyclic hydrocarbons and heterocyclic amines in relation to risk of colorectal neoplasia. Carcinogenesis 32: 203–209. 21081473 10.1093/carcin/bgq237 PMC3026844

[26] Yeh CC, Sung FC, Tang R, Chang-Chieh CR, Hsieh LL (2009) Polymorphisms of cytochrome P450 1A2 and N-acetyltransferase genes, meat consumption, and risk of colorectal cancer. Dis Colon Rectum 52: 104–111. 19273964 10.1007/DCR.0b013e31819734d7

[27] Saebø M, Skjelbred CF, Brekke Li K, Bowitz Lothe IM, Hagen PC, et al (2008) CYP1A2 164 A–>C polymorphism, cigarette smoking, consumption of well-done red meat and risk of developing colorectal adenomas and carcinomas. Anticancer Res 28: 2289–2295. 18751408

[28] Küry S, Buecher B, Robiou-du-Pont S, Scoul C, Sébille V, et al (2007) Combinations of cytochrome P450 gene polymorphisms enhancing the risk for sporadic colorectal cancer related to red meat consumption. Cancer Epidemiol Biomarkers Prev 16: 1460–1467. 17627011 10.1158/1055-9965.EPI-07-0236

[29] Yoshida K, Osawa K, Kasahara M, Miyaishi A, Nakanishi K, et al (2007) Association of CYP1A1, CYP1A2, GSTM1 and NAT2 gene polymorphisms with colorectal cancer and smoking. Asian Pac J Cancer Prev 8: 438–444. 18159984

[30] Bae SY, Choi SK, Kim KR, Park CS, Lee SK, et al (2006) Effects of genetic polymorphisms of MDR1, FMO3 and CYP1A2 on susceptibility to colorectal cancer in Koreans. Cancer Sci 97: 774–779. 16800822 10.1111/j.1349-7006.2006.00241.x PMC11160064

[31] Chen K, Jin MJ, Fan CH, Song L, Jiang QT, et al (2005) A case-control study on the association between genetic polymorphisms of metabolic enzymes and the risk of colorectal cancer. Zhonghua Liu Xing Bing Xue Za Zhi 26: 659–664. 16471212

[32] Landi S, Gemignani F, Moreno V, Gioia-Patricola L, Chabrier A, et al (2005) A comprehensive analysis of phase I and phase II metabolism gene polymorphisms and risk of colorectal cancer. Pharmacogenet Genomics 15: 535–546. 16006997 10.1097/01.fpc.0000165904.48994.3d

[33] Sachse C, Smith G, Wilkie MJ, Barrett JH, Waxman R, et al (2002) A pharmacogenetic study to investigate the role of dietary carcinogens in the etiology of colorectal cancer. Carcinogenesis 23: 1839–1849. 12419832 10.1093/carcin/23.11.1839

[34] Colditz GA, Burdick E, Mosteller F (1995) Heterogeneity in meta-analysis of data from epidemiologic studies: a commentary. Am J Epidemiol 142: 371–382. 7625401 10.1093/oxfordjournals.aje.a117644

[35] Higgins JP, Thompson SG, Deeks JJ, Altman DG (2003) Measuring inconsistency in meta-analyses. BMJ 327: 557–560. 12958120 10.1136/bmj.327.7414.557 PMC192859

[36] DerSimonian R, Laird N (1986) Meta-analysis in clinical trials. Control Clin Trials 7: 177–188. 3802833 10.1016/0197-2456(86)90046-2

[37] Mantel N, Haenszel W (1959) Statistical aspects of the analysis of data from retrospective studies of disease. J Natl Cancer Inst 22: 719–748. 13655060

[38] Egger M, Davey Smith G, Schneider M, Minder C (1997) Bias in meta-analysis detected by a simple, graphical test. BMJ 315: 629–634. 9310563 10.1136/bmj.315.7109.629 PMC2127453

[39] Boobis AR, Lynch AM, Murray S, de la Torre R, Solans A, et al (1994) CYP1A2-catalyzed conversion of dietary heterocyclic amines to their proximate carcinogens is their major route of metabolism in humans. Cancer Re 54: 89–94. 8261468

[40] Kootstra-Ros JE, Smallegoor W, van der Weide J (2005) The cytochrome P450 CYP1A2 genetic polymorphisms *1F and *1D do not affect clozapine clearance in a group of schizophrenic patients. Ann Clin Biochem 42: 216–219. 15949157 10.1258/0004563053857798

[41] van der Weide J, Steijns LS, van Weelden MJ (2003) The effect of smoking and cytochrome P450 CYP1A2 genetic polymorphism on clozapine clearance and dose requirement. Pharmacogenetics 13: 169–172. 12618594 10.1097/00008571-200303000-00006

[42] Nordmark A, Lundgren S, Ask B, Granath F, Rane A (2002) The effect of the CYP1A2*1F mutation on CYP1A2 inducibility in pregnant women. Br J Clin Pharmacol 54: 504–510. 12445029 10.1046/j.1365-2125.2002.01673.x PMC1874450

[43] Moonen HJ, Moonen EJ, Maas L Dallinga JW, Kleinjans JC, de Kok TM (2004) CYP1A2 and NAT2 genotype/phenotype relations and urinary excretion of 2-amino-1-methyl-6-phenylimidazo[45- b]pyridine (PhIP) in a human dietary intervention study. Food Chem Toxicol 42: 869–878. 15110095 10.1016/j.fct.2004.01.010

[44] Chen X, Wang L, Zhi L, Zhou G, Wang H, et al (2005) The G-113A polymorphism in CYP1A2 affects the caffeine metabolic ratio in a Chinese population. Clin Pharmacol Ther 78: 249–259. 16153396 10.1016/j.clpt.2005.05.012

[45] Takata K, Saruwatari J, Nakada N, Nakagawa M, Fukuda K, et al (2006) Phenotype-genotype analysis of CYP1A2 in Japanese patients receiving oral theophylline therapy. Eur J Clin Pharmacol 62: 23–28. 16385402 10.1007/s00228-005-0057-z

[46] Pavanello S, Pulliero A, Lupi S, Gregorio P, Clonfero E (2005) Influence of the genetic polymorphism in the 5′-noncoding region of the CYP1A2 gene on CYP1A2 phenotype and urinary mutagenicity in smokers. Mutat Res 587: 59–66. 16188490 10.1016/j.mrgentox.2005.08.008

[47] Wang H, Zhang Z, Han S, Lu Y, Feng F, et al (2012) CYP1A2 rs762551 polymorphism contributes to cancer susceptibility: a meta-analysis from 19 case-control studies. BMC Cancer 12: 528. 23157985 10.1186/1471-2407-12-528 PMC3526566

[48] Lohmueller KE, Pearce CL, Pike M, Lander ES, Hirschhorn JN (2003) Meta-analysis of genetic association studies supports a contribution of common variants to susceptibility to common disease. Nat Genet 33: 177–82. 12524541 10.1038/ng1071

[49] Morgan TM, Coffey CS, Krumholz HM (2003) Overestimation of genetic risks owing to small sample sizes in cardiovascular studies. Clin Genet 64: 7–17. 12791034 10.1034/j.1399-0004.2003.00088.x

