# Supplementary figures and images for: Quantitative Assessment of the Influence of Cytochrome P450 1A2 Gene Polymorphism and Colorectal Cancer Risk
Source: PLoS One. 2013 Aug 12;8(8):e71481. doi: 10.1371/journal.pone.0071481 (PMC3741149; doi:10.1371/journal.pone.0071481)

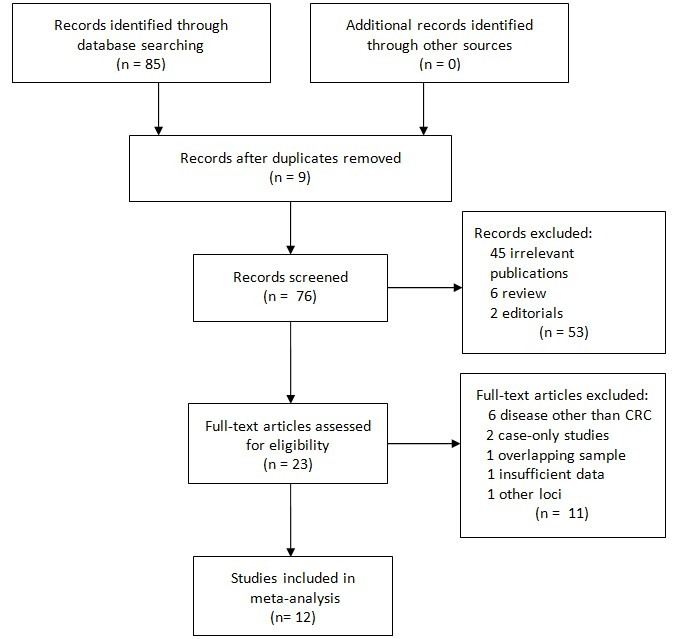

Supplement: Figure S1 — Flow chart of literature search for studies examining CYP1A2 gene polymorphism and risk of CRC. (TIF) [file pone.0071481.s001.tif]

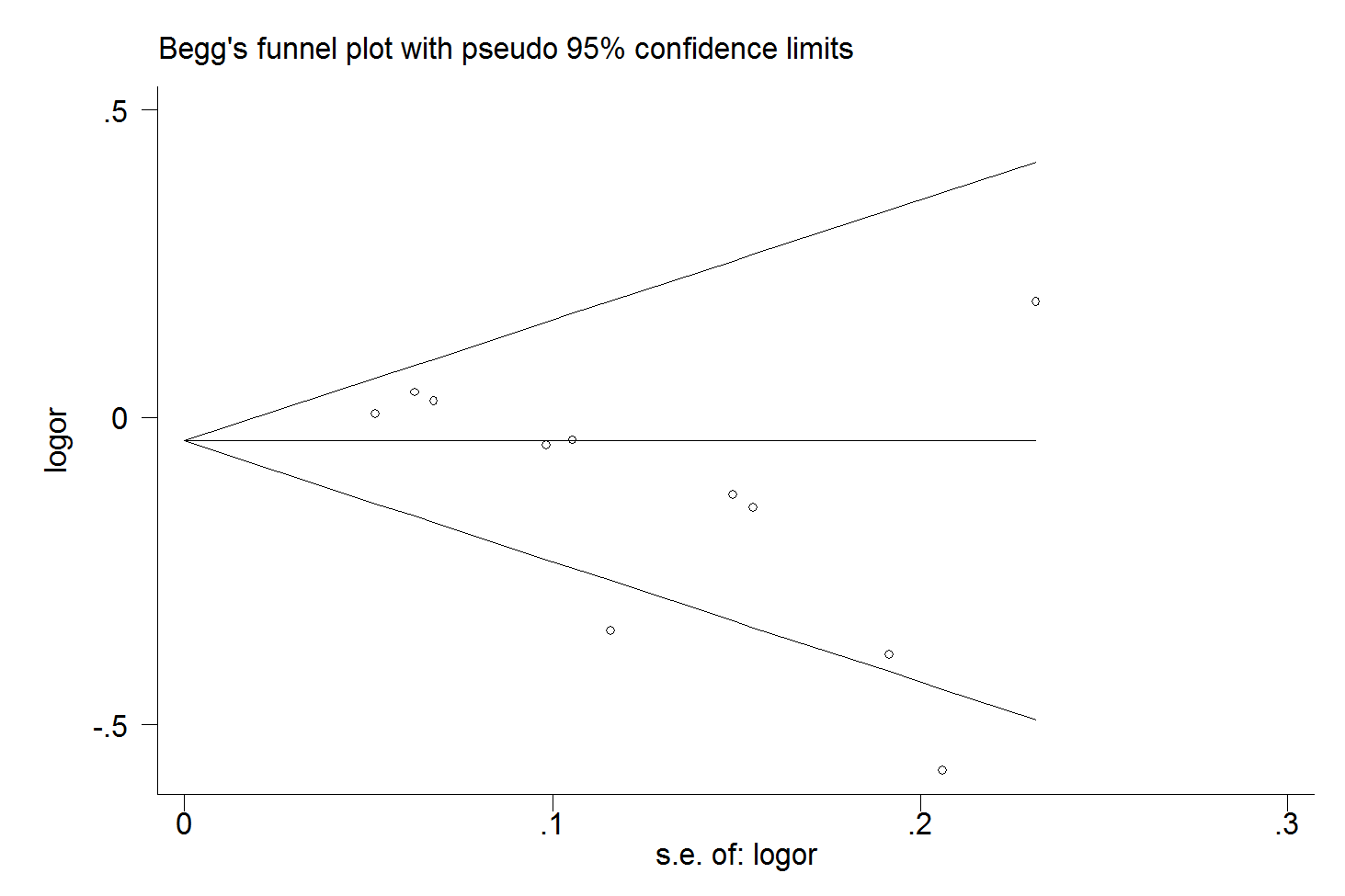

Supplement: Figure S2 — Funnel plot of studies of the CYP1A2 *F polymorphism and CRC showing a possible excess of smaller studies with strikingly positive findings beyond the 95% CI. (TIF) [file pone.0071481.s002.tif]

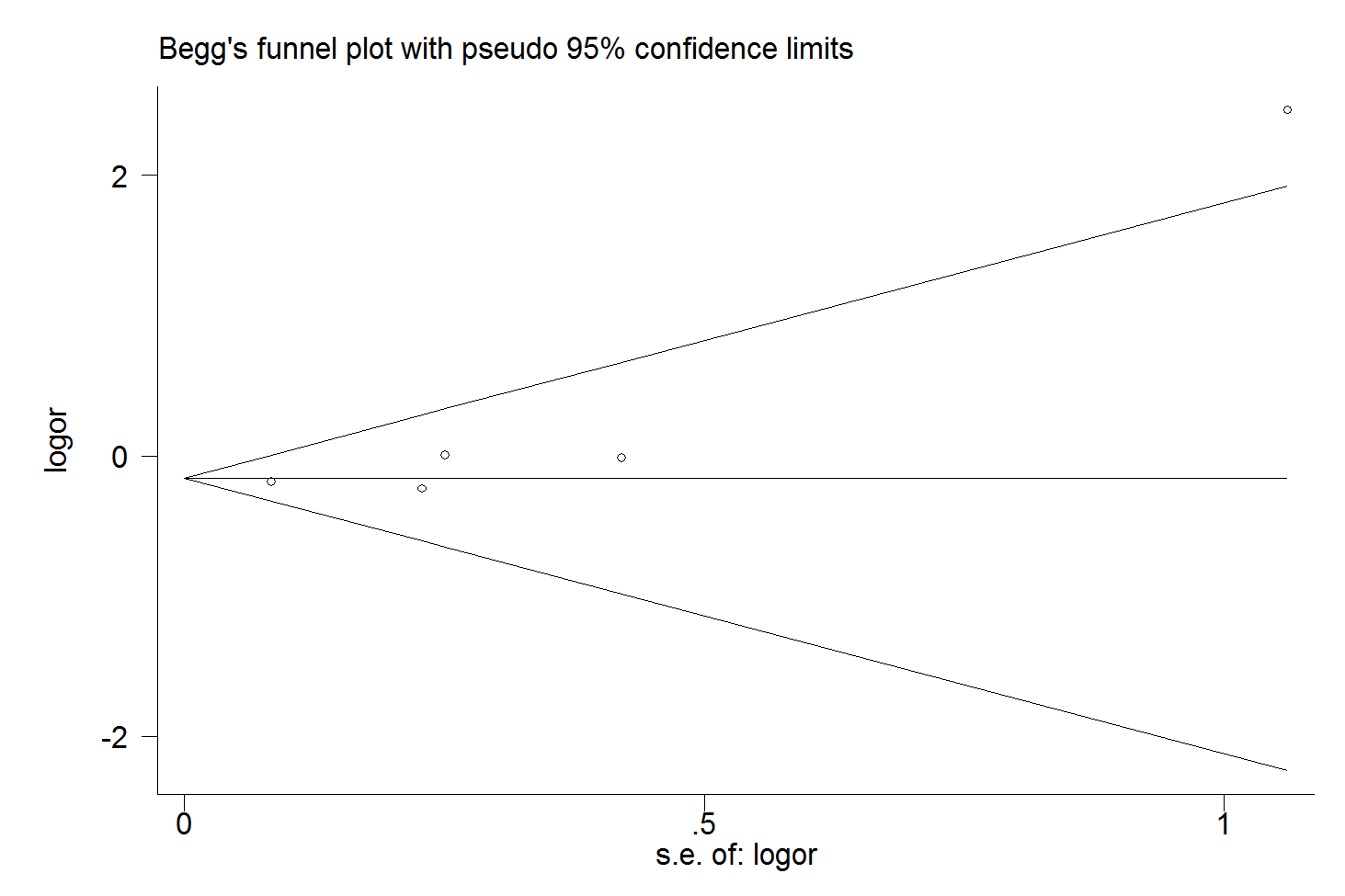

Supplement: Figure S3 — Funnel plot for the association between and CYP1A2 *C and CRC risk; Egger’s test was also performed to investigate the symmetry of the funnel plot ( P = 0.14). (TIF) [file pone.0071481.s003.tif]
